# Clinical Performance of the BD CTGCTV2 Assay for the BD MAX System for Detection of *Chlamydia trachomatis, Neisseria gonorrhoeae*, and *Trichomonas vaginalis* Infections

**DOI:** 10.1097/OLQ.0000000000001280

**Published:** 2020-09-09

**Authors:** Barbara Van Der Pol, Edith Torres-Chavolla, Salma Kodsi, Charles K. Cooper, Thomas E. Davis, Kenneth H. Fife, Stephanie N. Taylor, Michael H. Augenbraun, Charlotte A. Gaydos

**Affiliations:** From the ∗ Division of Infectious Diseases, University of Alabama at Birmingham School of Medicine, Birmingham, AL; † Becton, Dickinson and Company, BD Life Sciences—Diagnostic Systems, Sparks, MD; ‡ Sidney and Lois Eskenazi Hospital; § Indiana University School of Medicine, Indianapolis, IN; ¶ School of Medicine, Louisiana State University Health Sciences Center, New Orleans, LA; ∥ Division of Infectious Diseases, SUNY Downstate College of Medicine, Brooklyn, NY; ∗∗ Division of Infectious Diseases, Johns Hopkins University School of Medicine, Baltimore, MD

## Abstract

**Background:**

Diagnostic options to combat the increasing rates of sexually transmitted infections recorded throughout the world increasingly include multiplex assays. Here we describe the estimated sensitivity and specificity of a triplex molecular assay that simultaneously detects *Chlamydia trachomatis* (CT)*, Neisseria gonorrhoeae* (or gonococci [GC]), and *Trichomonas vaginalis* (TV).

**Methods:**

Participants (2547 women and 1159 men) were recruited from 12 clinics in the United States. BD CTGCTV2 for BD MAX System assay (CTGCTV2) results were obtained from vaginal and endocervical swabs, endocervical samples in cytology medium, and female and male urine. Results were compared with infection standards that were sample type and pathogen dependent.

**Results:**

Female specimen sensitivity estimates ranged from 92.7% to 98.4%, 92.9% to 100%, and 86.6% to 100% for CT, GC and TV, respectively. Male urine sensitivity estimates were 96.7%, 99.2%, and 97.9% for CT, GC, and TV, respectively. Specificity estimates were >98.7% for all sample types.

**Conclusions:**

BD CTGCTV2 performed well using a variety of sample types. As a true triplex assay, performed using a benchtop instrument, BD CTGCTV2 may be useful in settings where no testing is currently performed and in settings, such as reference laboratories, where testing turnaround time may be several days. Use of this assay at local laboratories may result in greater access to testing and a shorter time to result, which are important steps for improving our ability to combat sexually transmitted infections.

## Abstract 2

A study including female and male participants across 12 US clinics found that a triplex assay, BD CTGCTV2, performed on the benchtop BD MAX instrument, shows good performance for detection of *Chlamydia trachomatis*, *Neisseria gonorrhoeae*, and *Trichomonas vaginalis*.

Supplemental digital content is available in the text.

In the United States and across the world, rates of sexually transmitted infections (STIs) are increasingly prevalent.^1,2^ Infection with *Chlamydia trachomatis* (CT) and *Trichomonas vaginalis* (TV) remains treatable, but *Neisseria gonorrhoeae* (or gonococci [GC]) infections are becoming increasingly resistant to currently available antibiotics. Despite current control efforts, CT rates have increased by 25% since 2013, whereas GC rates have increased by 74%^2,3^ during the same period. Although we do not formally track TV cases in the United States, data^4–7^ from North America suggest that these infections are more common than gonococcal infections among women and that a large proportion, up to 70%,^8^ of male sexual partners of women with TV infections are also infected. The sequelae of untreated infections add substantial burden to the health care system^9–11^ because each has been associated with downstream consequences including pelvic inflammatory disease,^12,13^ tubal factor infertility,^14^ and adverse outcomes during pregnancy.^15–17^ Therefore, early detection and treatment are critical to stopping the spread and consequences of these infections, but the current paradigm is not achieving this goal.

One potential contributor toward our inability to control these infections may be the reduction in availability of STI-focused clinics resulting from cuts, beginning in 2013, in federal funding of STI-specific control programs. As a result, the proportion of infections that are identified at STI and family planning clinics has decreased over time,^2^ with more infections identified at clinics of unknown categories. Although this shift to receiving services at primary care clinics may be considered a positive change, the lack of impact seen as a result of this shift suggests that new solutions are needed that would facilitate routine local testing and screening consistent with professional guidelines and recommendations.^18,19^ These solutions should include diagnostics with rapid turnaround times and user-friendly processes that could normalize routine screening in primary care settings. The BD CTGCTV2 assay for BD MAX System (henceforth referred to as CTGCTV2) is a Food and Drug Administration–cleared, second generation of the molecular triplex assay, BD MAX CT/GC/TV, which has been previously described.^20,21^ CTGCTV2 has been Food and Drug Administration cleared for detection of TV DNA from male urine, and it includes an additional GC gene target (it now detects 2 GC genes that are required for a positive GC result). In addition, an increased volume size allows for multiple assays from a single swab specimen. Finally, CTGCTV2 is cleared for use with self-collected vaginal swabs. CTGCTV2 has the capacity to encourage testing in local venues that prefer not to send samples to centralized reference laboratories and thus eliminate sample transport times as a component of the time to results. In this study, we evaluated the performance of CTGCTV2 for simultaneous detection of CT, GC, and TV using samples obtained from both men and women.

## MATERIALS AND METHODS

### Population and Sample Collection

Men and women were recruited from 12 geographically diverse clinical sites in North America. Six sites enrolled women only, 5 sites enrolled women and men, and 1 site enrolled men only. Inclusion criteria included clinic attendees who were at least 18 years of age, or the minimum age allowed by local regulations, and for whom STI screening or diagnostic testing was appropriate. Women were ineligible if they had undergone a hysterectomy, had a cytology specimen collected in the previous 12 months, or were known to be more than 10 weeks pregnant. Men and women who had urinated in the previous hour were not eligible. Participants, 2547 women and 1159 men, were enrolled from sexually transmitted disease (STD), obstetrics/gynecology, family planning, and other clinics over a period of 11 months (July 2016–June 2017). Participants were categorized as symptomatic if they reported dysuria, urethral discharge, itching, odor, coital pain/difficulty/bleeding, testicular or scrotum pain/swelling, abnormal vaginal discharge, and/or pelvic/uterine/adnexal pain. All other participants were classified as asymptomatic.

To minimize the impact of collection order on assay performance, randomization occurred through rotation of the specimen collection order for the reference method and CTGCTV2 within each specimen type. Eight specimens were collected from each woman in the following order: 1 first-catch urine, 2 randomized patient-collected vaginal swab specimens, 2 randomized clinician-collected vaginal swab specimens, 2 randomized endocervical swab specimens, and 1 PreservCyt liquid-based cytology (LBC) specimen (collected using either the cervical broom or brush/spatula). One first-catch urine specimen was collected from each man in the study. Specimens from all compliant participants were tested using CTGCTV2 assay across 5 testing sites. Specimens intended for reference testing to determine the patient infection status (PIS) were handled in accordance with the instructions for use in the appropriate specimen collection kit package insert. The protocol was reviewed and approved by institutional review boards at each participating institution, and informed consent was obtained before sample collection. This report was prepared according to the Standards for Reporting of Diagnostic Accuracy.^22^

### Test Assay and PIS, and Reference Algorithm Assays

The CTGCTV2 assay is a CLIA–moderately complex assay that performs 1 to 24 samples per batch and requires approximately 15 minutes of hands-on time and slightly more than 3 hours from start to results.^21^ All amplified DNA targets and the Sample Processing Control, internal to each test, are detected using hydrolysis (TaqMan) probes labeled with different fluorophores. The CTGCTV2 assay uses 2 targets for CT, which are detected on the same optical channel because a positive reaction by either target is sufficient to call the sample positive. The targets cover regions on the chromosome as well as the plasmid to minimize the risk of loss of detection due to mutations. The assay also uses 2 targets for GC that are detected on 2 different optical channels, both of which are required to have a positive reaction to maximize specificity. CTGCTV2 uses one target for TV. The BD MAX System monitors the optical signals at the end of each cycle and interprets the data at the end of the reaction to provide qualitative test results for each analyte (i.e., positive, negative, incomplete, indeterminate, or unresolved).^23^

At the time of this study, there were no molecular assays that had claims for each of the sample types and analytes under evaluation, which precluded a head-to-head comparison within each specimen type. Furthermore, the number of swabs collected per female participant was limited and affected the assay/specimen type combinations that could be included in the study. Therefore, the reference assays used to define the PIS depended on the sample type claims for specific assays and included the following: Aptima Combo 2 (AC2; Hologic, San Diego, CA) used with LBC and urine specimens; Aptima TV (ATV; Hologic) performed on the Panther (ATV-P) and on the Tigris (ATV-T) platforms using vaginal swabs and urine specimens; ProbeTec CTQ^x^ and GCQ^x^ (CTQ/GCQ) and ProbeTec TVQ^x^ (TVQ) on the BD Viper System with XTR Technology (Viper XTR; BD Diagnostics, Sparks, MD) using endocervical swab and urine specimens for CT/GC and vaginal swabs for TV; and GeneXpert CT/GC and TV assays (CT/GCX and TVX; Cepheid, Sunnydale, CA) for urine specimens only.

**Figure 1:**
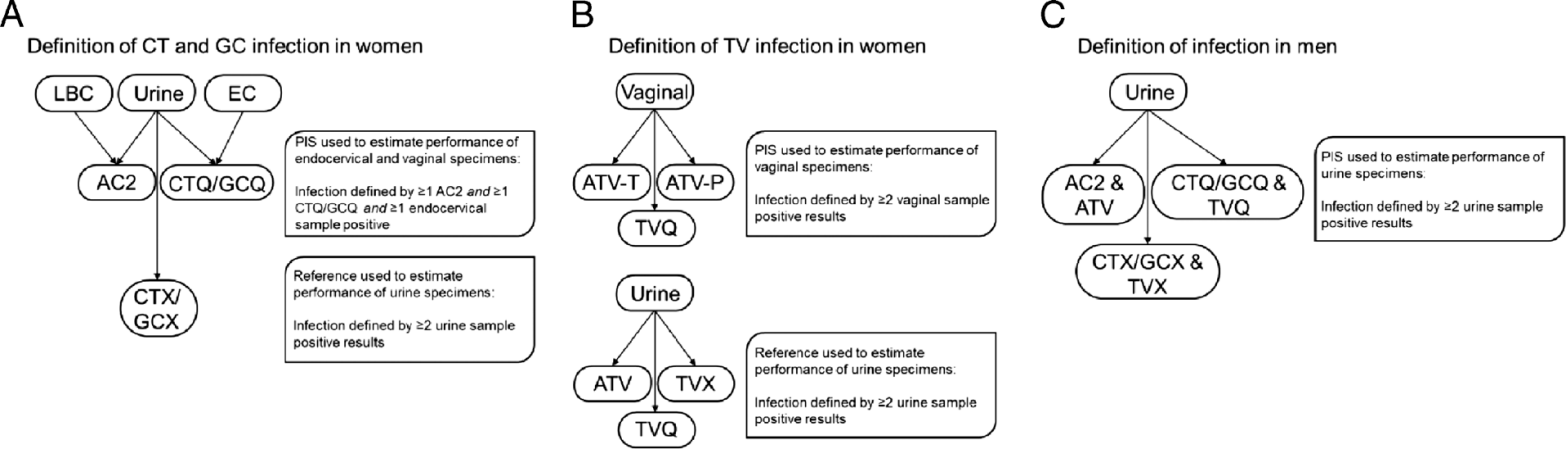
Definition of PIS in male and female participants. A, PIS for CT and GC in women using endocervical specimens and reference used to estimate performance of urine specimens. B, PIS for TV in women using vaginal specimens and reference used to estimate performance of urine specimens. C, PIS for CT, GC, and TV in men using urine specimens. EC, endocervical; AC2; APTIMA Combo 2; CTQ/GCQ/TVQ, BD ProbeTec CTQ, GCQ, and TVQ on Viper; CTX/GCX/TVX, GeneXpert CT/NG/TV; ATV-T and ATV-P, Aptima TV assay on Tigris and Panther systems, respectively.

For cervical (endocervical swabs or LBC) and vaginal performance, the CT and GC PIS was determined using a combination of results from cervical samples and urine specimens generated by the AC2 and CTQ/GCQ assays (Fig. 1A). Women were designated as infected if results were obtained from each of the 2 comparator assays with at least 1 positive result from a cervical specimen (i.e., 2 positive results only in urine were not categorized as infected for the cervical and vaginal performance analyses). The female PIS for TV was determined by testing vaginal swab specimens using ATV-P, ATV-T, and TVQ. Women were designated as TV-infected if at least 2 of 3 reference test results were positive (Fig. 1B). The clinical performance of the CTGCTV2 for the detection of any infections in male and female urine was calculated based on comparison to results from urine samples using 3 reference tests: AC2 or ATV, CTQ/GCQ or TVQ, and CTX/GCX and TVX. For all 3 organisms, urine samples were designated as positive if at least 2 of 3 reference nucleic acid amplification test (NAAT) results were positive (Fig. 1).

To evaluate the performance of the reference NAATs used in the reference algorithm within the clinical trial for CT and GC, a rotating PIS analysis was performed. In a study with multiple assay results, a rotating analysis calculates the performance of one test, with the remaining tests used as the reference method. According to the PIS reference algorithm for this study, a positive result was obtained when at least 2 assays were positive from a total of 4 reference NAAT results (2 from cervical specimens, 2 from urine specimens). For the rotating analysis, the reference NAAT was substituted by the new assay under evaluation (BD CTGCTV2). The rotating PIS analysis for TV was not performed because not all the reference tests were performed for all participants; only the specimens with discordant results between ATV-P/ATV-T and TVQ were tested with the opposite ATV assay to obtain the third result.

### Data Analysis

The Wilson score method was used to calculate 95% confidence intervals (CIs) for sensitivity and specificity estimates.^24^ To compare the performance characteristics between assays based on a rotating PIS approach, the Fisher exact test was performed and *P* values were reported.^25^ Rotating PIS analysis could be performed only for female urine and vaginal swabs and male urine, as these were the samples types tested by each of the comparator assays.

## RESULTS

From the 2547 women and 1159 men enrolled, 11 women and 10 men were excluded from the data analyses owing to enrollment issues including the following: patient withdrawal from study, duplicate enrollment, and inclusion criteria not met. The most common reasons for specimen exclusion included the following: missing specimens and/or reference test results, collection, transport/shipping, and/or processing errors. The CT PIS positivities were 5.5% (140/2534) in women and 13.4% (154/1146) in men. *N. gonorrhoeae* PIS positivities were 1.7% (43/2535) among women and 10.8% (124/1148) among men. The TV arm was terminated before the CT and GC arms because the target number of positive patients had been obtained (1765 women were included in the TV arm). Among women and men, the TV PIS positivities were 11.0% and 4.2%, respectively. The median age of women was 27 years (range of 18–68 years); the median age of men was 30 years (range of 18–75 years). Twenty-seven percent (27%) of women were enrolled from STD clinics, 25% from family planning clinics, 37% from obstetrics/gynecology clinics, and 12% from other clinical settings. For men, 96% of participants were enrolled from STD clinics (Table 1).

**TABLE 1:** Participants and Positivity Rate (PIS) by Clinic Type

Sex	Clinic Type	Evaluable Participants, % (n)	Positivity Rate (PIS) All Specimen Types Combined		
			*Chlamydia trachomatis*, % (n/N)	*Neisseria gonorrhoeae*, % (n/N)	*Trichomonas vaginalis*, % (n/N)
Female	Sexually transmitted disease/HIV	26.7 (676)	8.7 (59/675)	4.0 (27/676)	15.5 (60/386)
	Family planning	24.7 (626)	5.9 (37/625)	1.8 (11/625)	6.8 (29/424)
	Obstetrics and gynecology	36.9 (935)	3.6 (34/935)	0.4 (4/935)	6.7 (48/721)
	Other	11.8 (299)	3.3 (10/299)	0.3 (1/299)	24.9 (57/229)
	Overall	2536	5.5 (140/2534)	1.7 (43/2535)	11.0 (194/1760)
Male	Sexually transmitted disease/HIV	95.9 (1102)	13.3 (146/1099)	11.2 (123/1101)	4.4 (48/1100)
	Family planning	4.1 (47)	17.0 (8/47)	2.1 (1/47)	0.0 (0/47)
	Overall	1149	13.4 (154/1146)	10.8 (124/1148)	4.2 (48/1147)

PIS indicates patient infection status.

The initial assay nonreportable rate representing all targets, specimens, and types of nonreportable results combined was 2.4% (321/13,655; 95% CI, 2.1%–2.6%). Nonreportable results include the following: unresolved (invalid Sample Processing Control due to the presence of inhibitory samples or reagent failure), indeterminate (MAX instrument failure—with warning or error codes), and incomplete (incomplete run or aborted run by the operator). After a valid repeat test, 0.3% (38/13,649; 95% CI, 0.2%–0.4%) of specimens remained nonreportable and were excluded from the sensitivity and specificity statistical analysis. Final numbers for evaluable specimens included in the performance calculations are summarized in Table 2.

**TABLE 2:** CTCGTV2 Performance by Sample Type Compared with Reference Algorithm

Specimen Type	*Chlamydia trachomatis*, % [95% CI] (n/N)	*Neisseria gonorrhea*, % [95% CI] (n/N)	*Trichomonas vaginalis*, % [95% CI] (n/N)
Vaginal swab clinician-collected			
Sensitivity	98.4 [94.5–99.6] (126/128)	97.7 [87.9–99.6] (42/43)	97.8 [94.6–99.2] (182/186)
Specificity	98.9 [98.4–99.3] (2348/2374)	99.9 [99.6–100] (2457/2460)	99.6 [99.2–99.8] (1540/1546)
Vaginal swab self-collected			
Sensitivity	98.4 [94.5–99.6] (126/128)	100 [91.8–100] (43/43)	97.9 [94.7–99.2] (185/189)
Specificity	98.7 [98.1–99.0] (2348/2380)	99.8 [99.6–99.9] (2459/2463)	99.2 [98.6–99.5] (1540/1553)
Endocervical swab*			
Sensitivity	94.5 [89.1–97.3] (121/128)	95.3 [84.5–98.7] (41/43)	89.9 [84.8–93.5] (170/189)
Specificity	99.2 [98.8–99.5] (2366/2384)	100 [99.8–100] (2467/2468)	99.8 [99.4–99.9] (1547/1550)
PreservCyt LBC*			
Sensitivity	92.7 [86.8–96.1] (115/124)	92.9 [81.0–97.5] (39/42)	86.6 [80.9–90.7] (161/186)
Specificity	99.8 [99.5–99.9] (2340/2345)	100 [99.8–100] (2427/2428)	99.8 [99.4–99.9] (1507/1510)
Female urine†			
PPA	98.4 [94.3–99.6] (121/123)	100 [91.0–100] (39/39)	100 [97.8–100] (173/173)
NPA	99.3 [98.9–99.6] (2278/2293)	100 [99.8–100] (2379/2380)	99.6 [99.1–99.8] (1467/1473)
Male urine			
Sensitivity	96.7 [92.6–98.6] (148/153)	99.2 [95.5–99.9] (122/123)	97.9 [89.1–99.6] (47/48)
Specificity	99.4 [98.7–99.7] (981/987)	99.9 [99.4–100] (1018/1019)	99.7 [99.2–99.9] (1090/1093)

*Product use should be in accordance with product labeling. Refer to regional package insert for product claims and performance.

†Positive Percent Agreement and Negative Percent Agreement according to the 2/3 Composite Comparator Algorithm.

LBC indicates liquid-based cytology; NPA, negative percent agreement; PPA, positive percent agreement.

For CT using female specimens, the CTGCTV2 assay sensitivity ranged between 92.7% and 98.4%, depending on sample type, and the specificity ranged between 98.7% and 99.8% (Table 2). For male urine sensitivity and specificity, estimates were 96.7% and 99.4%, respectively, for CT. The positive predictive values (PPVs) based on observed CT prevalence (5.1% female, 13.4% male) were ≥79.6% for all female specimen types and 96.1% for male urine. *N. gonorrhoeae* detection sensitivity ranged between 92.9% and 100% and specificity ranged between 99.8% and 100% for female specimens. Using male urine, the sensitivity and specificity estimates were 99.2% and 99.9%, respectively. The PPVs based on observed GC prevalence (1.7% female, 10.8% male) were ≥91.4% for all female specimen types and 99.2% for male urine. Finally, for detection of TV, the assay sensitivity ranged from 86.6% to 97.8% and specificity ranged from 99.2% to 99.8% for specimens from women, whereas sensitivity and specificity estimates for male urine were 97.9% and 99.7%, respectively. The PPVs based on observed TV prevalence (10.8% female, 10.4% male) were ≥93.4% for all female specimen types and 94.0% for male urine.

**Figure 2:**
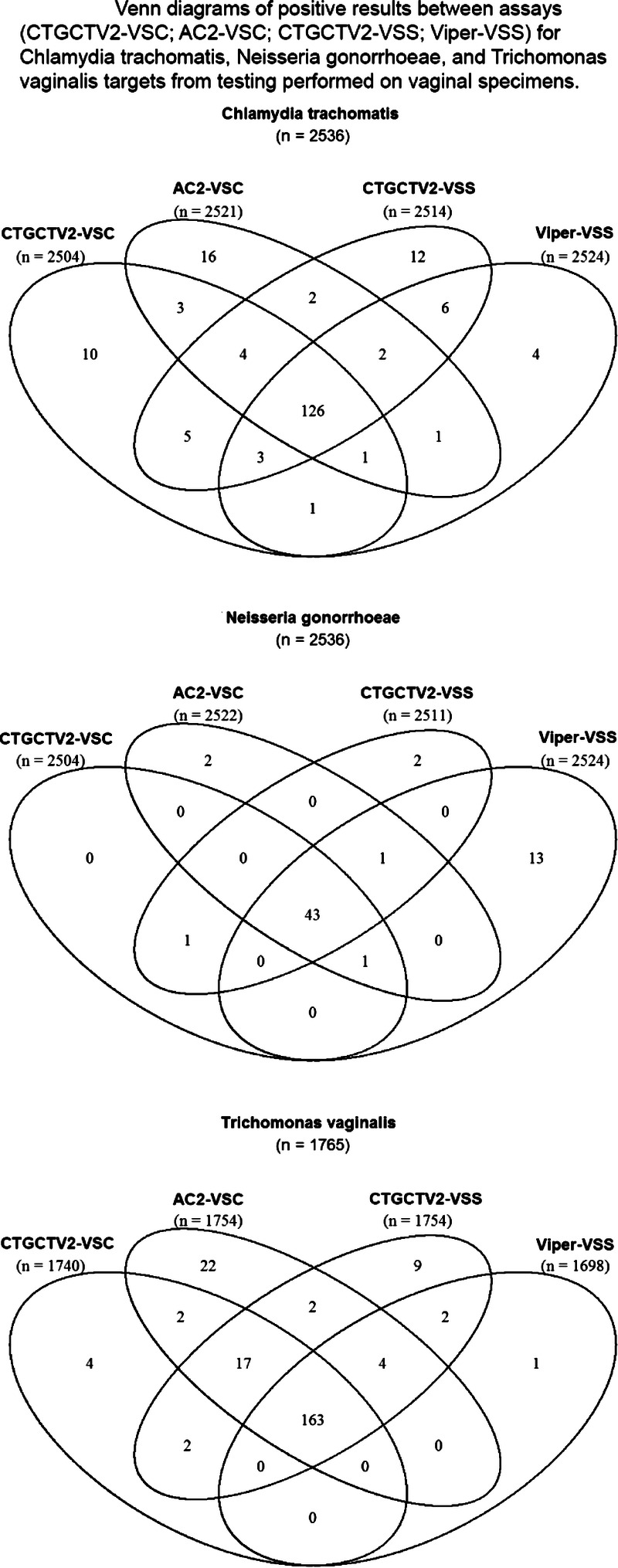
Venn diagrams of positive results between assays (CTGCTV2-VSC, AC2-VSC, CTGCTV2-VSS, Viper-VSS) for CT, GC, and TV targets from testing performed on vaginal specimens. CTGCTV2, BD CTGCTV2 assay for BD MAX; AC2, Aptima Combo 2; Viper, BD ProbeTec CTQ, GCQ, and TVQ on Viper; VSC, vaginal swab clinician-collected; VSS, vaginal swab self-collected.

**Figure 3:**
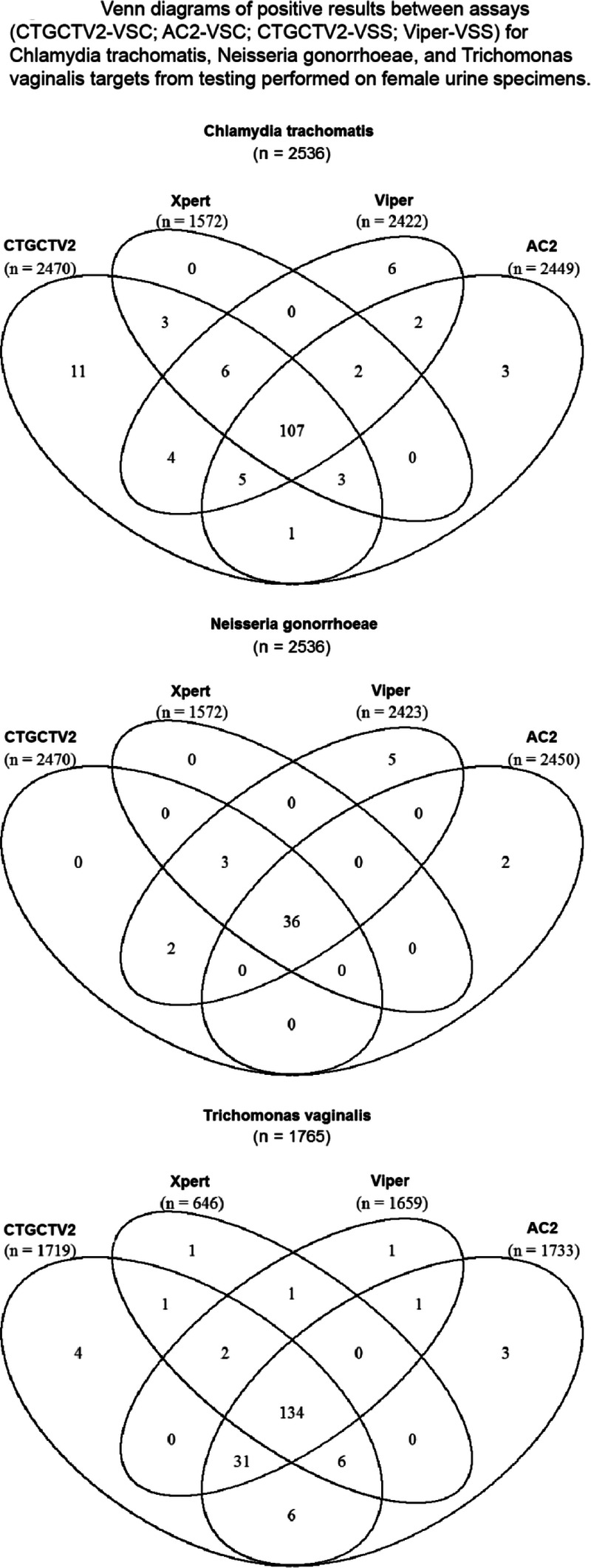
Venn diagrams of positive results between assays (CTGCTV2-VSC, AC2-VSC, CTGCTV2-VSS, Viper-VSS) for CT, GC, and TV targets from testing performed on female urine specimens. CTGCTV2, BD CTGCTV2 assay for BD MAX; AC2, Aptima Combo 2; Viper, BD ProbeTec CTQ, GCQ, and TVQ on Viper.

**Figure 4:**
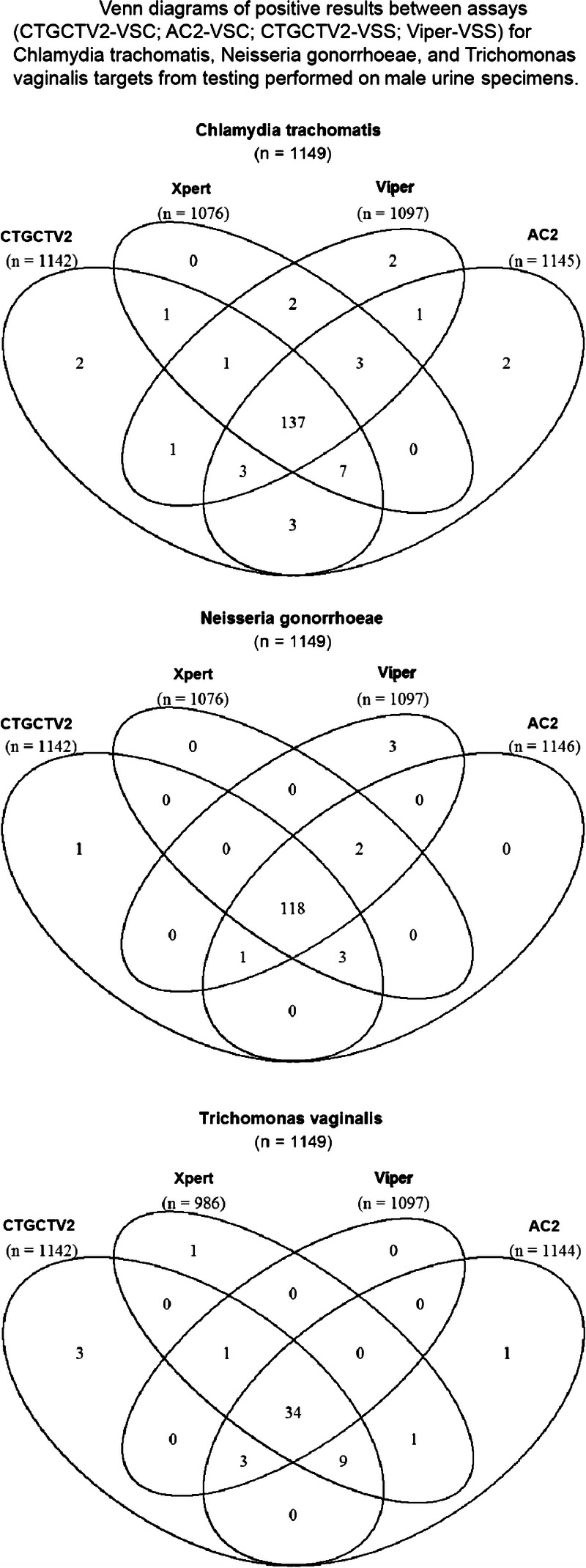
Venn diagrams of positive results between assays (CTGCTV2-VSC, AC2-VSC, CTGCTV2-VSS, Viper-VSS) for CT, GC, and TV targets from testing performed on male urine specimens. CTGCTV2, BD CTGCTV2 assay for BD MAX; AC2, Aptima Combo 2; Viper, BD ProbeTec CTQ, GCQ, and TVQ on Viper.

In addition to comparison of CTGCTV2 to predefined reference algorithms to determine performance characteristics related to detection of CT and GC, a rotating PIS analysis was conducted to estimate the performance all CT and GC assays used for testing with specimens obtained during this study. Table 3 summarizes the sensitivity and specificity for CT and GC using the rotating PIS analysis to evaluate the reference NAATs used in the study. The observed performance values of the predicate NAATs were compared with those obtained by CTGCTV2. No statistical difference was detected between any of the CT sensitivity or specificity estimates for the assays used for testing on vaginal swabs and female urine. In addition, no statistical difference was detected between any of the GC sensitivity estimates between the assays used for testing on vaginal swabs and female urine. For GC specificity estimates, group statistical analyses for vaginal (*P* = 0.0061) and urine (*P* = 0.0259) specimens revealed that at least one result was different from the others. Based on pairwise comparisons for GC specificity estimates from vaginal specimens, the Viper CT/GC (self-collected) had a significantly lower specificity estimate compared with the CTGTTV2 (clinician-collected) assay (*P* = 0.0450). Pairwise comparison for GC specificity from urine specimens revealed no significant differences between any of the assays (despite the significant difference revealed from group analysis). The distribution of positive results from vaginal swabs, female urine, and male urine samples is shown in Figures 2, 3, and 4, respectively. Similar data are shown for other specimen types in Supplemental Figure 1, http://links.lww.com/OLQ/A549.

**TABLE 3:** Female *Chlamydia trachomatis* and *Neisseria gonorrhoeae* Rotating Patient Infection Status* (Nucleic Acid Amplification Test From Endocervical/Urine Specimens)

	*Chlamydia trachomatis*; % [95% CI] (n/N)†,‡		*Neisseria gonorrhoeae*; % [95% CI] (n/N)§,¶	
	Sensitivity	Specificity	Sensitivity	Specificity
Vaginal specimens				
AC2-clinician∥	96.2 [91.4–98.4] (126/131)	98.8 [98.3–99.2] (2353/2382)	100 [91.8–100] (43/43)	99.8 [99.6–99.9] (2468/2472)
CT/GC Viper-Self**	96.2 [91.3–98.7] (125/130)	99.3 [98.6–99.6] (2,370/2,387)	100 [91.6–100] (42/42)	99.4 [99.0–99.7] (2,460/2,475)
CTGCTV2-clinician	98.4 [94.5–99.6] (126/128)	98.9 [98.4–99.3] (2,348/2,374)	97.7 [87.9–99.6] (42/43)	99.9 [99.6–100] (2,457/2,460)
CTGCTV2-self	98.4 [94.5–99.6] (126/128)	98.7 [98.1–99] (2,348/2,380)	100 [91.8–100] (43/43)	99.8 [99.6–99.9] (2,459/2,463)
Urine specimens				
AC2∥	85.6 [78.8–90.5] (119/139)	99.8 [99.6–99.9] (2288/2292)	88.1 [75.0–94.8] (37/42)	100 [99.8–100] (2390/2391)
CT/GC Viper**	88.5 [82.1–92.8] (123/139)	99.6 [99.3–99.8] (2267/2275)	97.6 [87.7–99.6] (41/42)	99.8 [99.5–99.9] (2368/2373)
CT/NG Xpert††	92.2 [86.2–95.7] (118/128)	99.8 [99.4–99.9] (1439/1442)	95.1 [83.9–98.7] (39/41)	100 [99.7–100] (1529/1529)
CTGCTV2	92.6 [86.9–95.9] (125/135)	99.4 [99.0–99.6] (2314/2328)	95.3 [84.5–98.7] (41/43)	100 [99.8–100.0] (2419/2419)

*Rotating PIS positive definition: At least 2 reference CT or GC NAATs results are positive, with at least 1 positive from each assay and at least 1 endocervical or LBC result positive for swab specimens.

†Statistical comparison by Fisher exact test revealed no significant difference in CT sensitivity or specificity values (*P* = 0.4967 and *P* = 0.1545, respectively) on vaginal specimens for the following tests: AC2, CT/GC Viper, CTGCTV2, and CTGCTV2 groups.

‡Statistical comparison by Fisher exact test revealed no significant difference in CT sensitivity or specificity values (*P* = 0.2048 and *P* = 0.0854, respectively) on urine specimens for the following tests: AC2, CT/GC Viper, CTGCTV2, and CTGCTV2 groups.

§Statistical comparison by Fisher exact test revealed no significant difference in GC sensitivity, but a significant difference between at least 2 groups for specificity values (*P* = 1.0000 and *P* = 0.0061, respectively) on vaginal specimens for the following tests: AC2, CT/GC Viper, CTGCTV2, and CTGCTV2 groups.

¶Statistical comparison by Fisher exact test revealed no significant difference in GC sensitivity, but a significant difference between at least 2 groups for specificity values (*P* = 0.3663 and *P* = 0.0259, respectively) on urine specimens for the following tests: AC2, CT/GC Viper, CTGCTV2, and CTGCTV2 groups.

∥Compared against references assays: CT/GC Viper Endocervical Swab and Urine, BD CTGCTV2 Endocervical Swab and Urine.

**Compared against references assays: AC2 LBC and Urine, BD CTGCTV2 Endocervical Swab and Urine.

††Compared against references assays: AC2 LBC and Urine, CT/GC Viper Endocervical Swab and Urine.

AC2 indicates Aptima Combo 2; CTGCTV2, BD CTGCTV2 assay for BD MAX; CT/GC Viper, BD ProbeTec CTQ^x^ and GCQ^x^ Viper; CT/NG Xpert, GeneXpert CT/NG.

## DISCUSSION

The CTGCTV2 assay, a true triplex assay, had excellent performance for all 3 sexually transmitted pathogens using a variety of specimen types. Similar to previous studies evaluating molecular assays for detection of STI,^26–30^ in this study, we demonstrated that vaginal swabs, whether collected by clinicians or patients, performed better than endocervical samples such as swabs or LBC. This reinforces the Centers for Disease Control and Prevention recommendation that vaginal swabs are the recommended sample type for all 3 pathogens. Furthermore, female urine performed well in this assay, which also supports noninvasive patient-collected sampling. Self-collection is more acceptable to women than sampling that requires a speculum-assisted pelvic examination. Given the lengthy period (3–5 years) now recommended between cervical cancer screening visits for women with negative human papillomavirus results, the capacity to perform annual sexual health screening will be highly dependent on the use on noninvasive sample collection.

The use of a PIS-based on multiple comparator tests can result in an underestimation of sensitivity, and thus, the performance estimates reported here may reflect the lower limit of the actual assay performance. This can be seen clearly when looking at the performance estimates in Table 3, which show a lower sensitivity for several tests than the true performance, which has been established in many other studies. The AC2 performance is a case in point with sensitivity estimates substantially lower than actual performance. This is often the result of comparing DNA-based tests to RNA-based tests rather than a reflection of true sensitivity of the assay.

The previous version of this assay did not include claims for use with male urine for detection of trichomonas, nor were there claims for use of PreservCyt LBC or clinician-collected vaginal swabs. This new formulation allows clinician to choose from a wide variety of the most commonly used sample types and perform a convenient triplex test that does not require multiple orders and tests from the same sample. The addition of male urine for TV testing is a step forward because in this study, more than 4% of men were infected with trichomonas. Although this positivity rate was not as high as the rates for chlamydia or gonorrhea, this still represents a significant disease burden. Using a triplex assay to automatically generate CT, GC, and TV results will facilitate appropriate treatment, including treatment of TV from a single visit rather than waiting for men to fail treatment and then test for TV, as is common practice in many settings.

This study was limited in the ability to collect multiple specimens so that all female sample types could be tested using all comparator assays. This was due to (1) the need to minimize patient burden and (2) the lack of claims for all sample types and all pathogens on each comparator platform. As a result, the PIS was complicated and may have led to bias in the performance estimations. However, the use of at least 3 assays, although not always the same 3, for each sample type comparison was a rigorous study design. This also highlights the uniqueness of the CTGCTV2 assay: this assay can simultaneously detect all 3 pathogens across a wide range of sample types. There remains a need to evaluate this platform for performance using anorectal and oropharyngeal sample types, but such studies are underway. Finally, it is possible that coinfection (CT/GC, CT/TV, and GC/TV) might impact the performance of CTGCTV2 compared with that associated with a single infection. However, all 5 targets (2 CT, 2 GC, and 1 TV) for the CTGCTV2 assay have dedicated detection wavelengths, which reduces the chance of signal crossover and subsequently should reduce the occurrence of false-negative results that could occur because of preferential amplification of one target over another during assay performance. In addition, each of the analytes was tested near the limit of detection in the presence of high levels of other analytes to demonstrate that the risk of nondetection for any of the targets in the presence of mixed infections is low. Overall coinfection rates were low in this study, occurring at a rate of 1.0% of the study population (data not shown); this is similar to rates reported for coinfection with these organisms in the United States, previously.^31^

In summary, the CTGCTV2 assay has advantages over the previous version and has advantages over assays that require separate testing for CT/GC and TV. The use of a benchtop instrument that can perform midrange volumes of testing and the ability to use a wide variety of genital sample types will make this assay a good fit in settings that want to test locally which could improve STI screening coverage in many settings.

## Supplementary Material

SUPPLEMENTARY MATERIAL
