# Supplementary material for: Clinical Performance of the BD CTGCTV2 Assay for the BD MAX System for Detection of Chlamydia trachomatis, Neisseria gonorrhoeae, and Trichomonas vaginalis Infections
Source: Sex Transm Dis. 2020 Sep 9;48(2):134–40. doi: 10.1097/OLQ.0000000000001280 (PMC7817187; doi:10.1097/OLQ.0000000000001280)
Supplement: SUPPLEMENTARY MATERIAL [file olq-48-134-s001.docx]

## SUPPLEMENTAL TABLES

## TABLE S1

| **Table S1.** *Chlamydia trachomatis*, *Neisseria gonorrhoeae*, and *Trichomonas vaginalis* overall positive predictive value and negative predictive value based on observed prevalence and reference algorithm, by gender and specimen type | | | | |
| --- | --- | --- | --- | --- |
|  |  |  | *Chlamydia trachomatis* | |
| Gender | Sample type | Prevalence | PPV (95% CI) | NPV (95% CI) |
| Female | VCS | 5.1 | 82.7 (76.6-87.5) | 99.9 (99.7-100.0) |
|  | VSS | 5.1 | 79.6 (73.5-84.6) | 99.9 (99.7-100.0) |
|  | ES | 5.1 | 87.0 (80.9-91.3) | 99.7(99.4-99.9) |
|  | LBC | 5.1 | 95.9 (90.8-98.2) | 99.6(99.3-99.8)) |
|  | Urine | 5.1 | 88.9 (82.9-93.0) | 99.9(99.7-100) |
| Male | Urine | 13.4 | 96.1 (91.9-98.2) | 99.5(98.9-99.8) |
|  |  |  |  |  |
|  |  |  | *Neisseria gonorrhoeae* | |
| Gender | Sample type | Prevalence | PPV (95% CI) | NPV (95% CI) |
| Female | VCS | 1.7 | 93.3 (82.5-97.6) | 100 (99.8-100.0) |
|  | VSS | 1.7 | 91.4 (80.6-96.5) | 100 (99.9-100.0) |
|  | ES | 1.7 | 97.6 (87.8-99.6) | 99.9 (99.7-100.0) |
|  | LBC | 1.7 | 97.5 (87.3-99.5) | 99.9 (99.7-100.0) |
|  | Urine | 1.6 | 97.4 (87.1-99.5) | 100 (99.9-100) |
| Male | Urine | 10.8 | 99.2 (95.6-99.9) | 99.9 (99.5-100) |
|  |  |  |  |  |
|  |  |  | *Trichomonas vaginalis* | |
| Gender | Sample type | Prevalence | PPV (95% CI) | NPV (95% CI) |
| Female | VCS | 10.8 | 96.8 (93.3-98.5) | 99.7 (99.3-99.9) |
|  | VSS | 10.8 | 93.4 (89.2-96.0) | 99.7 (99.4-99.9) |
|  | ES | 10.8 | 98.3 (95.0-99.4) | 98.8 (98.2-99.2) |
|  | LBC | 10.8 | 98.1 (94.7-99.4) | 98.4 (97.7-98.9) |
|  | Urine | 10.4 | 96.6 (92.9-98.4) | 100 (99.7-100) |
| Male | Urine | 4.2 | 94.0 (84.2-97.9) | 99.9 (99.5-100) |
| **Abbreviations:** PPV, positive predictive value; NPV, negative predictive value; VSC, Vaginal swab Clinician-collected; VSS, vaginal swab self-collected; ES, endocervical swab; LBC, PreservCyt | | | | |

## TABLE S2

| ***Table S2.*** *Male Chlamydia trachomatis/ Neisseria gonorrhoeae /Trichomonas vaginalis rotating* patient infection status***^a^*** *(*nucleic acid amplification test *from urine specimens)* | | | |
| --- | --- | --- | --- |
| **Specimen type** | *Chlamydia trachomatis*; % [95% CI] (n/N) | *Neisseria gonorrhoeae*; % [95% CI] (n/N) | *Trichomonas vaginalis*; % [95% CI] (n/N) |
| **Aptima^b,c^** |  |  |  |
| Sensitivity | 97.4 [93.5-99.0] (150/154) | 100 [97.0-100] (124/124) | 100 [92.3-100] (46/46) |
| Specificity | 99.4 [98.7-99.7] (983/989) | 100 [99.6-100] (1021/1021) | 99.8 [99.3-99.9] (1093/1095) |
| **Viper^d,e^** |  |  |  |
| Sensitivity | 98.0 [94.2-99.3] (144/147) | 100 [96.9-100] (121/121) | 82.6 [69.3-90.9] (38/46) |
| Specificity | 99.4 [98.6-99.7] (944/950) | 99.7 [99.1-99.9] (973/976) | 100 [99.6-100] (1050/1050) |
| **Xpert^f,g^** |  |  |  |
| Sensitivity | 96.1 [91.8-98.2] (148/154) | 100 [97.0-100] (123/123) | 97.8 [88.4-99.6] (44/45) |
| Specificity | 99.8 [99.2-99.9] (918/920) | 100 [99.6-100] (952/952) | 99.8 [99.2-99.9] (938/940) |
| **CTGCTV2** |  |  |  |
| Sensitivity | 96.7 [92.6-98.6] (148/153) | 99.2 [95.5-99.9] (122/123) | 97.9 [89.1-99.6] (47/48) |
| Specificity | 99.4 [98.7-99.7] (981/987) | 99.9 [99.4-100] (1018/1019 ) | 99.7 [99.2-99.9] (1090/1093) |
| **Abbreviations:**Aptima, Aptima TV Assay; Viper, BD ProbeTec TV^x^ Viper; Xpert, Gene Xpert TVX; CTGCTV2, BD CTGCTV2 assay for BD MAX | | | |
|  | | | |
| **^a^**Rotating PIS positive definition: At least two out of three results on urine samples  **^b^**Includes either AC2 (CT and GC) or ATV (TV)  **^c^**Compared against references assays on Viper, Xpert, and the CTGCTV2 assay  **^d^**Includes either CT/GCQ (CT and GC) or TVQ (TV)  **^e^**Compared against references assays on Aptima, Xpert, and the CTGCTV2 assay  **^f^**Includes either CT/GCX (CT and GC) or TVX (TV)  **^g^**Compared against references assays on Aptima, Viper, and the CTGCTV2 assay | | | |
